# Comparison of diffusing capacity of carbon monoxide (DLCO) and total lung capacity (TLC) between Indigenous Australians and Australian Caucasian adults

**DOI:** 10.1371/journal.pone.0248900

**Published:** 2021-04-02

**Authors:** Timothy Howarth, Helmi Ben Saad, Ara J. Perez, Charmain B. Atos, Elisha White, Subash S. Heraganahally

**Affiliations:** 1 College of Health and Human Sciences, Charles Darwin University, Darwin, Northern Territory, Australia; 2 Darwin Respiratory and Sleep Health, Darwin Private Hospital, Darwin, Northern Territory, Australia; 3 Université de Sousse, Faculté de Médecine de Sousse, Laboratoire de Physiologie, Sousse, Tunisia; 4 Department of Physiology and Functional Exploration, Farhat HACHED Hospital, Sousse, Tunisia; 5 Heart Failure Research Laboratory (LR12SP09), Farhat HACHED Hospital, Sousse, Tunisia; 6 Department of Respiratory and Sleep Medicine, Royal Darwin Hospital, Darwin, Northern Territory, Australia; 7 College of Medicine and Public Health and Northern Territory Medical Programme, Flinders University, Adelaide, South Australia, Australia; University of Calcutta, INDIA

## Abstract

**Background and objective:**

Currently there is paucity of evidence in the literature in relation to normative values for diffusing capacity of carbon monoxide (DLCO) and total lung capacity (TLC) among Indigenous Australians. Hence, in this study we assessed the DLCO and TLC parameters among Indigenous Australians in comparison to Australian Caucasian counterparts.

**Methods:**

DLCO and TLC values were assessed and compared between Indigenous Australians and Australian Caucasians matched for age, sex and body mass index, with normal chest radiology.

**Results:**

Of the 1350 and 5634 pulmonary function tests assessed in Indigenous Australian and Australian Caucasian adults respectively, a total of 129 Indigenous Australians and 197 Australian Caucasians met the inclusion criteria. Absolute DLCO and TLC values for Indigenous Australians were a mean 4.3 ml/min/mmHg (95% CI 2.86, 5.74) and 1.03 L (95% CI 0.78, 1.27) lower than Australian Caucasians (p<0.01). Percentage predicted values were 15.38 (95% CI 11.59, 19.17) and 16.63 (95% CI 13.59, 19.68) points lower for DLCO and TLC, respectively. Lower limit of normal (LLN) values did not significantly differ between groups, however a significantly greater proportion of Indigenous Australians recorded values below the LLN in comparison to Australian Caucasians for DLCO (64 vs. 25%, p<0.01) and TLC (66 vs. 21%, p<0.01). Significant differences for the interaction of sex on DLCO and TLC were noted in Australian Caucasians, with reduced or absent sex differentiation among Indigenous Australians.

**Conclusions:**

There are significant differences in DLCO and TLC parameters between Indigenous Australian compared to Australian Caucasians. Appropriate DLCO and TLC norms need to be established for Indigenous Australians.

## Introduction

Indigenous Australians experience a significantly higher burden of respiratory disease in comparison to their non-Indigenous peers [1–4]. Multiple factors underpin this high burden of respiratory disease in this population, from higher rates of premature birth and low birth weight [5], to childhood infections [6] and higher rates of smoking [7, 8]. Currently there is scarcity of published data for adult reference values of normative lung function parameters (LFPs) among Indigenous Australians [9, 10]. Recent work has sought to address this gap in the literature with reports demonstrating significantly reduced Forced Vital Capacity (FVC) and Forced Expiratory Volume in one second (FEV_1_), yet typically nearly preserved FEV_1_/FVC ratio [11–13]. Moreover, a recent study from our centre found the Global Lung function Initiative (GLI-2012) norms do not fit for Indigenous Australian adults, regardless of which ethnicity option was selected, including “others/mixed” [14]. Normative reference values for diffusing capacity of carbon monoxide (DLCO) and total lung capacity (TLC) have also yet to be established within the Indigenous Australian population [15].

DLCO, a measure of alveolar surface area and diffusion capacity, appears to be a more sensitive measure of morbidity in comparison to FVC or FEV_1_ [16]. FVC and/or FEV_1_ may remain relatively stable over time even in the presence of advancing pulmonary disease and worsening respiratory symptoms, while DLCO could demonstrate significant decline/abnormality [17, 18]. Furthermore, DLCO has a significantly greater correlation to radiographic indices of pulmonary disease than any other pulmonary function test (PFT) parameters [19]. This factor may be particularly important in resource poor settings, such as those experienced by many Indigenous Australians as access to radiography is extremely limited among patients living in regional and remote communities. DLCO (expressed as a percentage of normative reference values) is among the preoperative tests performed before undergoing pulmonary surgical interventions (**eg**, lobectomy up to a pneumonectomy). In practice, any patient free from cardiovascular risk factors, with a DLCO >80% (in addition to a FEV_1_ >80%) can undergo surgical pulmonary interventions, such as pneumonectomy without further investigation–thus it is critical normative reference values actually reflect normative values within the population being tested [20].

In a similar vein, accurate reference values of TLC have at least three factors of primary care significance. First, it permits the determination of the TLC limits of normal (**eg**: lower limit of normal (LLN)), and therefore facilitates accurate diagnosis of specific respiratory disease patterns (**ie**; restrictive ventilatory defect (TLC < LLN) [21] and lung hyperinflation (TLC > upper limit of normal) [22]. Second, TLC allows more meaningful interpretations of FEV_1_ and FVC [23]. Third, reduced TLC (***eg***; TLC < LLN) correlates with both morbidity & mortality, quality of life and physical activity [24, 25]. Variations in TLC may be a function of intrinsic factors (body stature and body composition distribution) within the Indigenous Australian population. However, significant extrinsic barriers also co-exist throughout the life course in this population, such as maternal smoking [26], lower birthweight and gestational age [27], infections through infancy and childhood [6] and higher adolescent and adulthood smoking rates [28], which may contribute to impaired lung growth and development, translating to reduced adult TLC values. Hence, it may be reasonable to document the normative DLCO and TLC parameters in this adult Indigenous Australian population. Therefore, the aim of this study was to evaluate the DLCO and TLC values among radiographically healthy Indigenous Australians and compare these to Australian Caucasians residing in the Top End Health Service (TEHS) region of the Northern Territory (NT) of Australia.

## Methods

### Setting and ethical approval

This retrospective study was conducted at the Respiratory and Sleep service based at the Royal Darwin Hospital and Darwin Respiratory and Sleep Health, Darwin Private Hospital in the TEHS region, NT of Australia. According to the Australian Bureau of Statistics, about 3.3% of the Australian population identify as Indigenous Australians [29, 30]. In the NT, about 30% of the population identify as Indigenous Australian, the highest proportion amongst all Australian states and territories. This study was approved by the Human Research Ethics Committee of the NT, TEHS and Menzies School of Health Research. (Reference no: HREC 2019–3445). The HREC advised specific written/oral consent was not required as data was routinely collected and analysed anonymously.

### Study participants

Study participants included were Indigenous Australian and Australian Caucasian adults living in the TEHS region, who underwent a pulmonary function test between 2012 and 2020 and were referred for PFTs by a primary health practitioner, respiratory specialist or other specialist physician as a part of routine clinical care. A large part of the methodology of this study has been described in our earlier report [14].

### Australian Caucasian subject

During the study period, 5634 PFTs were performed on self-identified Australian Caucasian adults. For comparative analysis, subjects who had normal chest radiology were included. The original database was searched for Australian Caucasians to be matched as close as possible for sex, age ranges (18–25, 25–35, 35–55 & 55+ years) and corpulence status (underweight (body mass index (BMI) ≤ 18.5 kg/m^2^), normal weight (BMI 18.6–24.9 kg/m^2^), overweight (BMI 25.0–29.9 kg/m^2^), obese (BMI ≥ 30.0 kg/m^2^)) against Indigenous Australian counterparts.

### PFTs

All PFTs including DLCO and TLC were performed according to the American thoracic and the European respiratory societies guidelines/recommendations, including calibration of equipment and quality assurance [16, 31] and as detailed in our previous reports [11, 14]. For measuring TLC and DLCO, the portable single-breath diffusing capacity device (EasyOne Pro®, ndd Medical Technologies) was used [32, 33]. Only PFTs graded as acceptable for session quality were included in this study. For acceptable quality for DLCO testing inspiratory volume >85% best vital capacity was used. TLC was measured by single breath manoeuvre technique [34]. Limitations to single-breath TLC measurement in the presence of airway obstruction are corrected for in EasyOne Pro device using Punjabi et al.’s equation [35]. In our centre and in the absence of specific respiratory reference values for the Indigenous Australian population, the predicted normative values for TLC and DLCO were calculated using the National Health and Nutrition Examination Survey Caucasian reference set’s (no ethnic correction was used) [36]. The following parameters were determined: TLC sb (single breath) (L, %), and DLCO (ml/min/mmHg, %). For the purpose of this study only DLCO and TLC were included in the analysis. No correctional factors for haemoglobin were applied, hence DLCO adj (adjusted) values and carbon monoxide transfer coefficient (= DLCO/alveolar volume) were not included in the analysis.

### Clinical data collection and study subject inclusion/exclusion criteria

As per standard protocol, participants age (on the date of testing), sex, height and weight were recorded. Smoking history was recorded to identify current-, past- or never- smokers, and to quantify the pack years of smoking [37]. Participants who underwent PFTs and had chest radiology (Chest X-Ray and or computed tomography chest) performed within a calendar year of the PFT date were initially included in the study, to assess the presence/absence of any radiological pulmonary abnormalities. Only participants with normal chest radiology were included for analysis.

### Statistical methods

Continuous parameters were tested for normality with the Shapiro Wilks distribution test. Age, height, weight, BMI (weight/height^2^), DLCO and TLC were found to deviate only slightly from normal distribution, however smoking pack years deviated to a significant extent and thus was treated as non-parametric. Non-parametric parameters were presented as medians (interquartile ranges), normally distributed parameters as means (95% confidence intervals (CIs)), and categorical parameters as numbers (%). Demographic parameters were compared between Indigenous Australian and Australian Caucasian adults with two-tailed proportions z-tests for categorical parameters, two-tailed students t-test for parameters approximating normal distribution and equality of medians test for the non-parametric. DLCO and TLC absolute and predicted values were tested against each other by age (≤ 35 & > 35 years), sex and Indigenous status using unpaired, two-sided, students t-tests. The proportion of participants outside of norms (**ie**, < LLN) for DLCO and TLC were tested by age, sex and Indigenous status using two-tailed proportions z-tests. Kernel density plots utilising Epanechnikov kernels were produced for each age (≤ 35 & > 35 years), sex (female & male) and Indigenous strata to demonstrate differences in distribution of DLCO and TLC. Multivariate linear regression models were developed to define the effect of known significant factors (age, sex, height, weight and smoking status) alongside Indigenous status on absolute DLCO and TLC, reporting beta coefficients (95% CIs), factor p-values, model p-values and adjusted Pearson’s determination coefficient “r^2^”. All data were analysed in STATA IC 15 (StataCorp, Texas) and alpha was set to 0.05 throughout.

## Results

### Baseline clinical parameters

A total of 326 subjects (129 Indigenous Australian (80 females) and 197 Australian Caucasians (135 females)) were included. Indigenous Australian participants were significantly younger than Australian Caucasians (p = 0.01) (Table 1). BMI did not significantly differ between the two populations (p = 0.96), but a significantly higher proportion of Indigenous Australians were in the underweight category (7 vs. 1%, p<0.01) and a lower though not significantly proportion in the overweight category (25 vs. 35%, p = 0.063). A significantly higher proportion of Indigenous Australians reported current smoking (55 vs. 22%, p<0.01), however reported pack years did not differ by Indigenous status (13 (4, 26) vs. 15 (5, 30), p = 0.77).

**Table 1:** Demographics and anthropometrics of Indigenous Australian and Australian Caucasians participants (n = 326).

		Indigenous Australian n = 129	Australian Caucasians n = 197	p-value
**Sex (female)**		80 (62%)	135 (69%)	0.23
**Age (years)**		46.22 (44.05, 48.39)	49.64 (48.07, 51.21)	0.01*
**Age ranges (years)**	**< 25**	8 (6%)	1 (1%)	<0.01*
	**25 ≤ 35**	20 (16%)	21 (11%)	0.27
	**35 ≤ 55**	66 (51%)	104 (53%)	0.88
	**> 55**	35 (27%)	71 (36%)	0.067
**Height (m)**		1.66 (1.64, 1.67)	1.68 (1.67, 1.69)	0.044*
**Weight (kg)**		84.08 (80, 88.16)	86.1 (83.19, 89)	0.41
**Body mass index (BMI, kg/m**^**2**^**)**		30.51 (29.09, 31.93)	30.47 (29.53, 31.4)	0.96
**Corpulence status**	**Underweight**	9 (7%)	2 (1%)	<0.01*
	**Normal weight**	24 (19%)	30 (15%)	0.42
	**Overweight**	32 (25%)	68 (35%)	0.063
	**Obese**	64 (50%)	97 (49%)	0.95
**Smoking status**	**Non-smoker**	23 (18%)	67 (34%)	<0.01*
	**Former smoker**	34 (27%)	87 (44%)	<0.01*
	**Current smoker**	70 (55%)	43 (22%)	<0.01*
**Pack years** **†**		13 (4, 26)	15 (5.3, 30)	0.77

Data were expressed as mean (95% confidence interval) and number (%).

Differences between groups tested with two-tailed students T-test for continuous parameters and proportions z-test for categorical parameters.

†Pack years data were available for 97 & 118 Indigenous Australian and Australian Caucasian subjects, respectively.

†This variable was non-parametrically distributed, thus displayed as median (interquartile range) and tested via equality of medians test.

*Denote statistically significant differences (p<0.05).

### DLCO data

Indigenous Australian participants displayed significantly reduced absolute DLCO values, with a mean difference of 4.3 ml/min/mmHg (2.86, 5.74, p<0.01) (Table 2). Percentage predicted values were a mean 15.38 (11.59, 19.17, p<0.01) percentage points lower. After being stratified by age and sex, differences were noted in the effect of Indigenous status between strata. Where for females the mean differences by Indigenous status were 11.44 (-2.17, 25.05) and 13.95 (9.15, 18.75) percentage points for under and over 35 years respectively, for males they were 21.33 (6.1, 36.56) and 21.33 (13.45, 29.23).

**Table 2:** Diffusing capacity of carbon monoxide (DLCO) values for participants by Indigenous status, stratified by sex and age (≤ 35 & > 35 years).

	Indigenous Australian (n = 129)	Australian Caucasians (n = 197)	p-value
**Total sample**			
**DLCO (ml/min/mm/Hg)**	18.75 (17.65, 19.85)	23.05 (22.13, 23.97)	<0.01*
**DLCO (%)**	71 (68, 74)	86 (84, 89)	<0.01*
**DLCO LLN**	20.16 (19.45, 20.86)	20.4 (19.82, 20.99)	0.99
**DLCO < LLN**	82 (64%)	49 (25%)	<0.01*
**Females**			
**≤ 35 years**	**n = 14 (18%)**	**n = 11 (9%)**	
**DLCO (ml/min/mm/Hg)**	20.23 (17.59, 22.87)	25.87 (21.79, 29.95)	<0.01*
**DLCO (%)**	78.29 (70.27, 86.3)	89.73 (76.93, 102.53)	0.10
**DLCO LLN**	20.19 (18.76, 21.63)	23.03 (22.07, 23.99)	<0.01*
**DLCO < LLN**	7 (50%)	4 (36%)	0.50
**> 35 years**	**n = 66 (82%)**	**n = 124 (91%)**	
**DLCO (ml/min/mm/Hg)**	16.47 (15.32, 17.61)	20.03 (19.3, 20.76)	<0.01*
**DLCO (%)**	70.21 (66.05, 74.38)	84.16 (81.42, 86.9)	<0.01*
**DLCO LLN**	17.7 (17.05, 18.35)	18.1 (17.68, 18.53)	0.29
**DLCO < LLN**	42 (64%)	32 (26%)	<0.01*
**Males**			
**≤ 35 years**	**n = 14 (29%)**	**n = 11 (18%)**	
**DLCO (ml/min/mm/Hg)**	26.59 (22.13, 31.05)	33.72 (29.59, 37.84)	0.02*
**DLCO (%)**	77.21 (64.83, 89.6)	98.55 (89.79, 107.3)	0.01*
**DLCO LLN**	27.5 (26.3, 28.7)	27.14 (24.61, 29.66)	0.76
**DLCO < LLN**	9 (64%)	1 (9%)	0.01*
**> 35 years**	**n = 35 (71%)**	**n = 51 (82%)**	
**DLCO (ml/min/mm/Hg)**	19.33 (17.1, 21.57)	27.48 (25.64, 29.32)	<0.01*
**DLCO (%)**	67 (60.08, 73.92)	88.33 (83.68, 92.99)	<0.01*
**DLCO LLN**	21.83 (20.92, 22.74)	23.98 (22.97, 24.98)	<0.01*
**DLCO < LLN**	24 (69%)	12 (24%)	<0.01*

Abbreviations: DLCO, diffusing capacity of carbon monoxide; LLN, lower limit of normal.

Data were expressed as mean (95% confidence interval) and number (%).

DLCO (%, LLN) data were derived from the NHANES (National Health and Nutrition Examination Survey) norms.

Differences between groups tested with two-tailed students T-test for continuous parameters and proportions z-test for categorical parameters.

*Denote statistically significant differences (p<0.05).

Absolute LLN values for DLCO did not significantly differ across the total sample (p = 0.99), however were significantly reduced among Indigenous Australian females aged ≤ 35 years (p<0.01) and Indigenous Australian males aged > 35 years (p<0.01). However, a significantly greater proportion of Indigenous Australians both overall, and in each stratum aside from females ≤ 35 years, recorded DLCO values below the LLN (64 vs. 25%, p<0.01).

**Fig 1:**
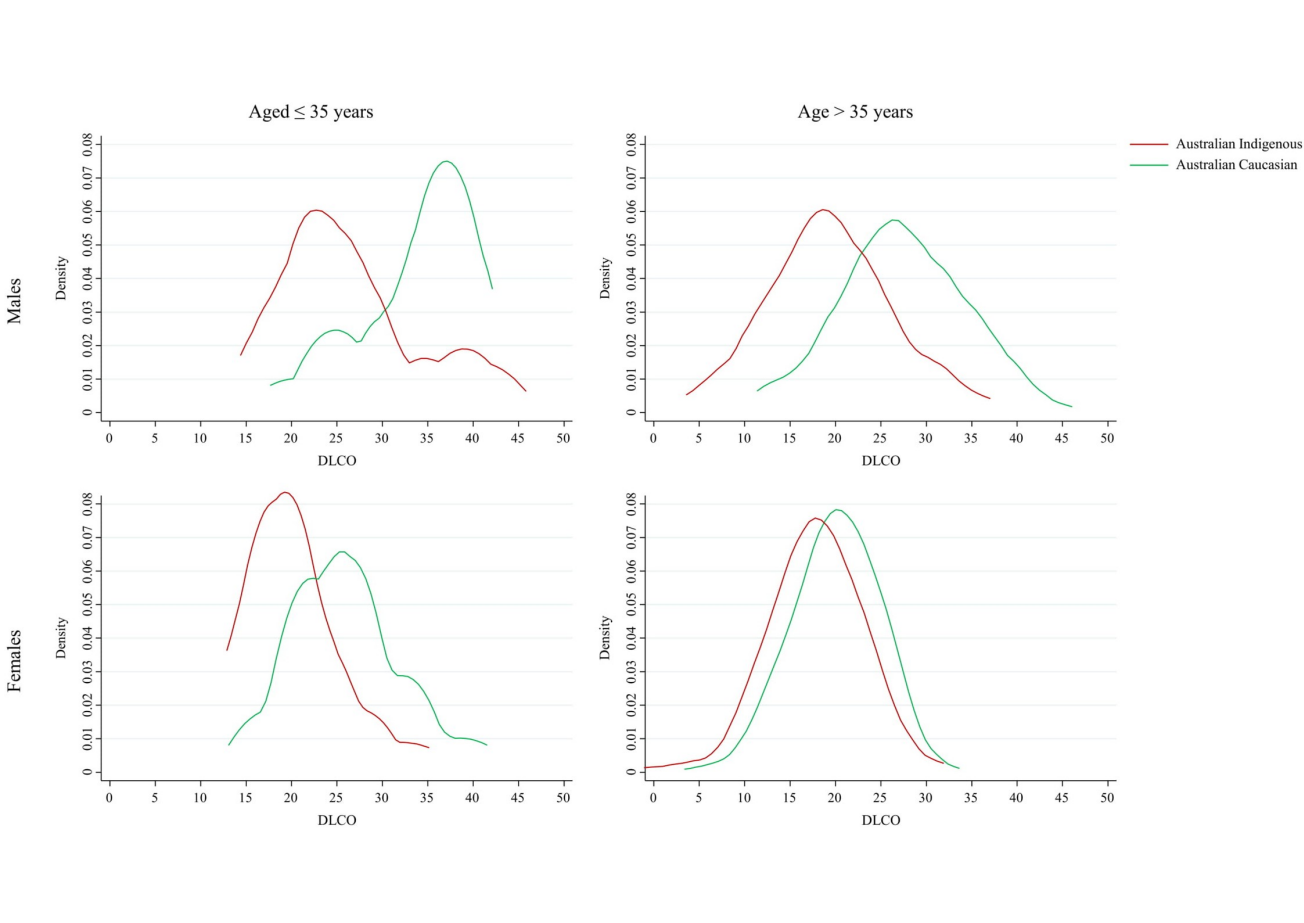
Kernel density plots of absolute diffusing capacity of carbon monoxide (DLCO) values by age group and sex. Bandwidth set at 3.00, Epanechnikov kernels.

The distribution of absolute DLCO scores followed a similar pattern for Indigenous Australian and Australian Caucasians for females (any age) and for males > 35 years, though displaced to lower values for Indigenous Australians. However, for males ≤ 35 years the distribution was flipped (Fig 1). Where Australian Caucasian participants displayed an extended tail toward low DLCO values, Indigenous Australians displayed a tail toward high DLCO values. Furthermore, the disparity in DLCO values by sex was significantly reduced among Indigenous Australians, particularly in those aged > 35 years.

**Fig 2:**
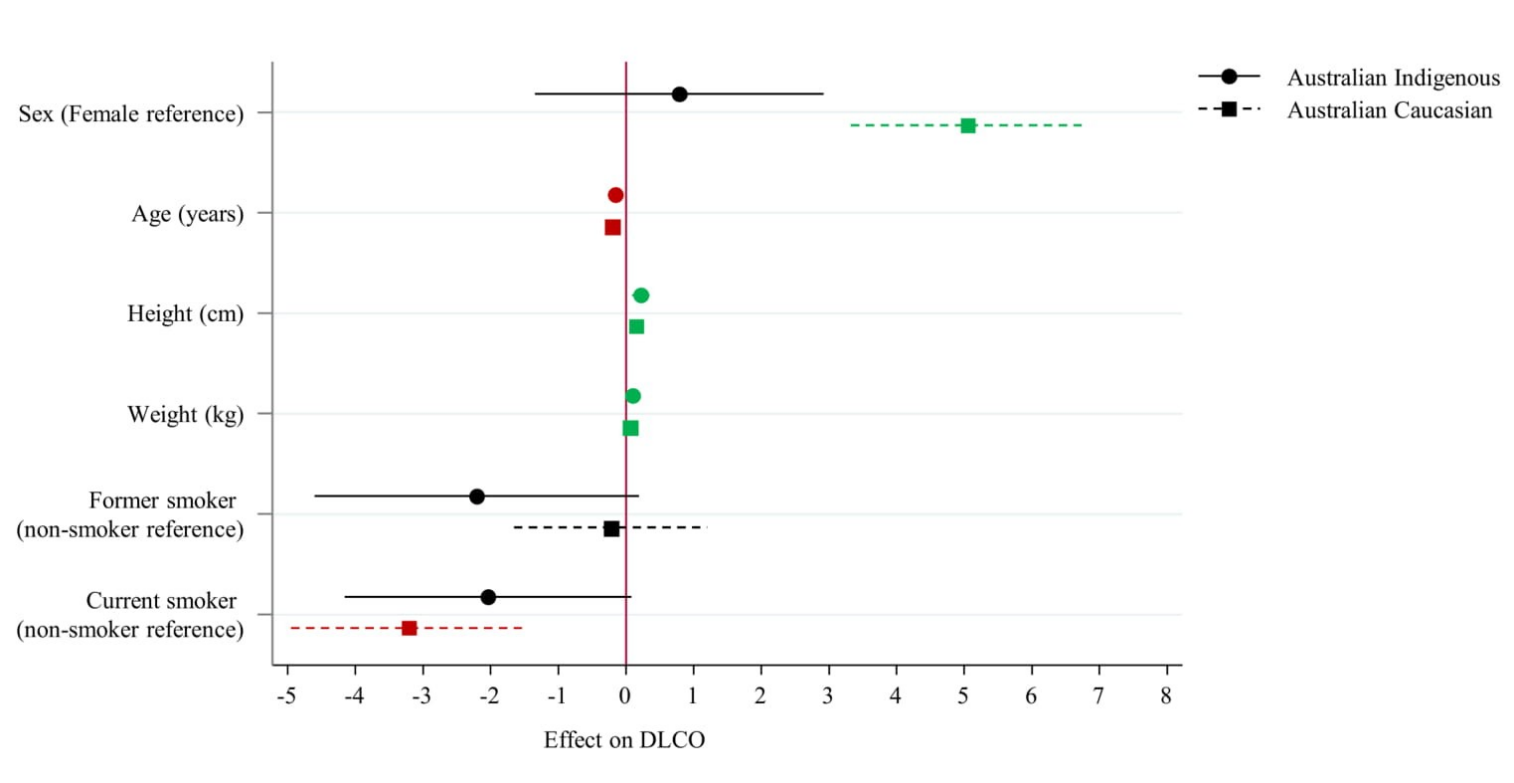
Coefficients plot of multivariate regression effects on diffusing capacity of carbon monoxide (DLCO).

In the development of the multivariate regression model, a significant interaction was noted between Indigenous status and sex, thus separate models were developed for Indigenous Australian and Australian Caucasians (Table 3). Significant differences are seen between the two models for multiple factors. Sex did not display any significant association within Indigenous Australian subjects (p = 0.47) whereas it did for Australian Caucasians (p<0.01). Former and current smoking did not reach significance for effect on DLCO values for Indigenous Australians, though current smoking did significantly negatively correlate in Australian Caucasians (p<0.01). The effect of advancing age differed notably between groups, as exhibited by the difference in confidence interval to mean by Indigenous status, with a larger effect visible in the Australian Caucasian group (Fig 2).

**Table 3:** Multivariate regression on diffusing capacity of carbon monoxide (DLCO) value outcome reporting coefficients (95% CIs).

	Indigenous Australian		Australian Caucasians	
	Beta (95% CIs)	p-value	Beta (95% CIs)	p-value
**Sex (Female reference)**	0.79 (-1.35, 2.92)	0.466	5.06 (3.32, 6.79)	<0.01
**Age (years)**	-0.16 (-0.22, -0.09)	<0.001	-0.21 (-0.27, -0.15)	<0.01
**Height (cm)**	0.21 (0.09, 0.35)	0.001	0.15 (0.06, 0.25)	<0.01
**Weight (kg)**	0.1 (0.06, 0.14)	<0.001	0.05 (0.02, 0.08)	<0.01
**Former smoker (non-smoker reference)**	-2.21 (-4.6, 0.19)	0.071	-0.23 (-1.66, 1.2)	0.75
**Current smoker (non-smoker reference)**	-2.04 (-4.16, 0.08)	0.059	-3.21 (-4.95, -1.46)	<0.01
**Model R**^**2**^ **& p-value**	0.515	<0.001	0.584	<0.01

Data were expressed as means (95% confidence interval).

R^2^: adjusted form of Pearson’s product moment correlation coefficient.

Values fit within the ’Moderate’ correlation range (0.5–0.7).

### TLC data

Indigenous Australian study participants displayed significantly reduced absolute TLC, with a mean difference of 1.03 L (0.78, 1.27, p<0.01) between groups (Table 4). Percentage predicted values were a mean 16.63 (11.59, 19.68, p<0.01) percentage points lower. When stratified by age and sex, differences were noted in the effect of Indigenous status between strata. Where for females the mean difference by Indigenous status increased with age from 8.88 (-2.08, 19.83) to 17.99 (13.71, 22.27) percentage points for under and over 35 years respectively, for males the mean difference decreased from 20.1 (9.56, 30.64) to 15.45 (10.0, 20.9).

**Table 4:** Total Lung Capacity (TLC) values for participants by Indigenous status and stratified by sex and age (≤ 35 & > 35 years).

	Indigenous Australian (n = 129)	Australian Caucasians (n = 197)	p-value
**Total sample**			
**TLC (L)**	4.06 (3.87, 4.25)	5.09 (4.93, 5.24)	<0.01*
**TLC (%)**	73.31 (70.62, 76)	89.94 (88.2, 91.69)	<0.01*
**TLC LLN**	4.51 (4.35, 4.67)	4.62 (4.48, 4.76)	0.35
**TLC < LLN**	85 (66%)	41 (21%)	<0.01*
**Females**			
**≤ 35 years**	**n = 14 (18%)**	**n = 11 (9%)**	
**TLC (L)**	4.09 (3.55, 4.63)	4.81 (4.31, 5.31)	0.048*
**TLC (%)**	81.21 (72.5, 89.92)	90.09 (83.32, 96.87)	0.11
**TLC LLN**	4.04 (3.76, 4.31)	4.33 (4.08, 4.58)	0.11
**TLC < LLN**	6 (43%)	2 (18%)	0.19
**> 35 years**	**n = 66 (82%)**	**n = 124 (91%)**	
**TLC (L)**	3.59 (3.38, 3.81)	4.57 (4.44, 4.7)	<0.01*
**TLC (%)**	73.14 (69.18, 77.1)	91.13 (88.79, 93.46)	<0.01*
**TLC LLN**	3.95 (3.8, 4.09)	4.01 (3.94, 4.09)	0.36
**TLC < LLN**	43 (65%)	18 (15%)	<0.01*
**Males**			
**≤ 35 years**	**n = 14 (29%)**	**n = 11 (18%)**	
**TLC (L)**	4.97 (4.37, 5.57)	6.44 (5.78, 7.1)	<0.01*
**TLC (%)**	71.36 (63.43, 79.28)	91.45 (84, 98.91)	<0.01*
**TLC LLN**	5.78 (5.48, 6.07)	5.88 (5.53, 6.23)	0.62
**TLC < LLN**	11 (79%)	2 (18%)	<0.01*
**> 35 years**	**n = 35 (71%)**	**n = 51 (82%)**	
**TLC (L)**	4.57 (4.21, 4.93)	6.11 (5.82, 6.4)	<0.01*
**TLC (%)**	71.26 (66.28, 76.23)	86.71 (83.63, 89.78)	<0.01*
**TLC LLN**	5.27 (5.06, 5.48)	5.87 (5.66, 6.08)	<0.01*
**TLC < LLN**	25 (71%)	19 (37%)	<0.01*

Abbreviations: TLC, Total lung capacity; LLN, Lower limit of normal.

Data were expressed as mean (95% confidence interval) and number (%).

TLC (%, LLN) data were derived from the NHANES (National Health and Nutrition Examination Survey) norms.

Differences between groups tested with two-tailed students T-test for continuous parameters and proportions z-test for categorical parameters.

*Denote statistically significant differences (p<0.05).

Absolute LLN values for TLC did not significantly differ across the total sample (p = 0.35), nor any strata aside from males aged > 35 years (p<0.01). However, a significantly greater proportion of Indigenous Australians both overall, and in each stratum aside from females ≤ 35 years, recorded TLC values below the LLN (66 vs. 21%, p<0.01).

**Fig 3:**
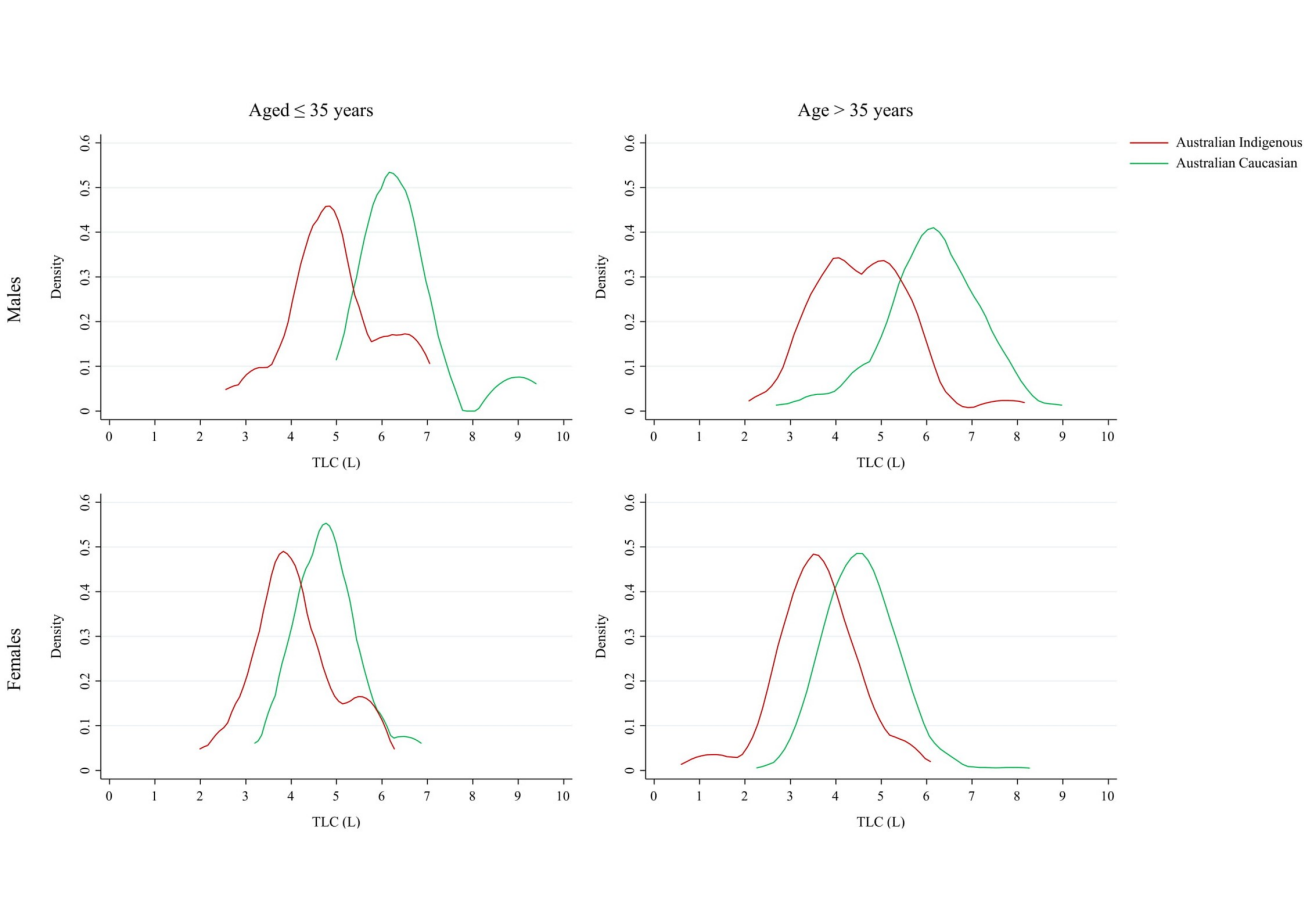
Kernel density plots of absolute Total Lung Capacity (TLC) values by age group and sex. Bandwidth set at 0.400, Epanechnikov kernels.

The distribution of absolute TLC scores followed a similar pattern for Indigenous Australian and Australian Caucasians females and males (any age), though displaced to lower values for Indigenous Australians (Fig 3). The disparity in TLC values by sex was significantly reduced among Indigenous Australians compared to Australian Caucasians.

**Fig 4:**
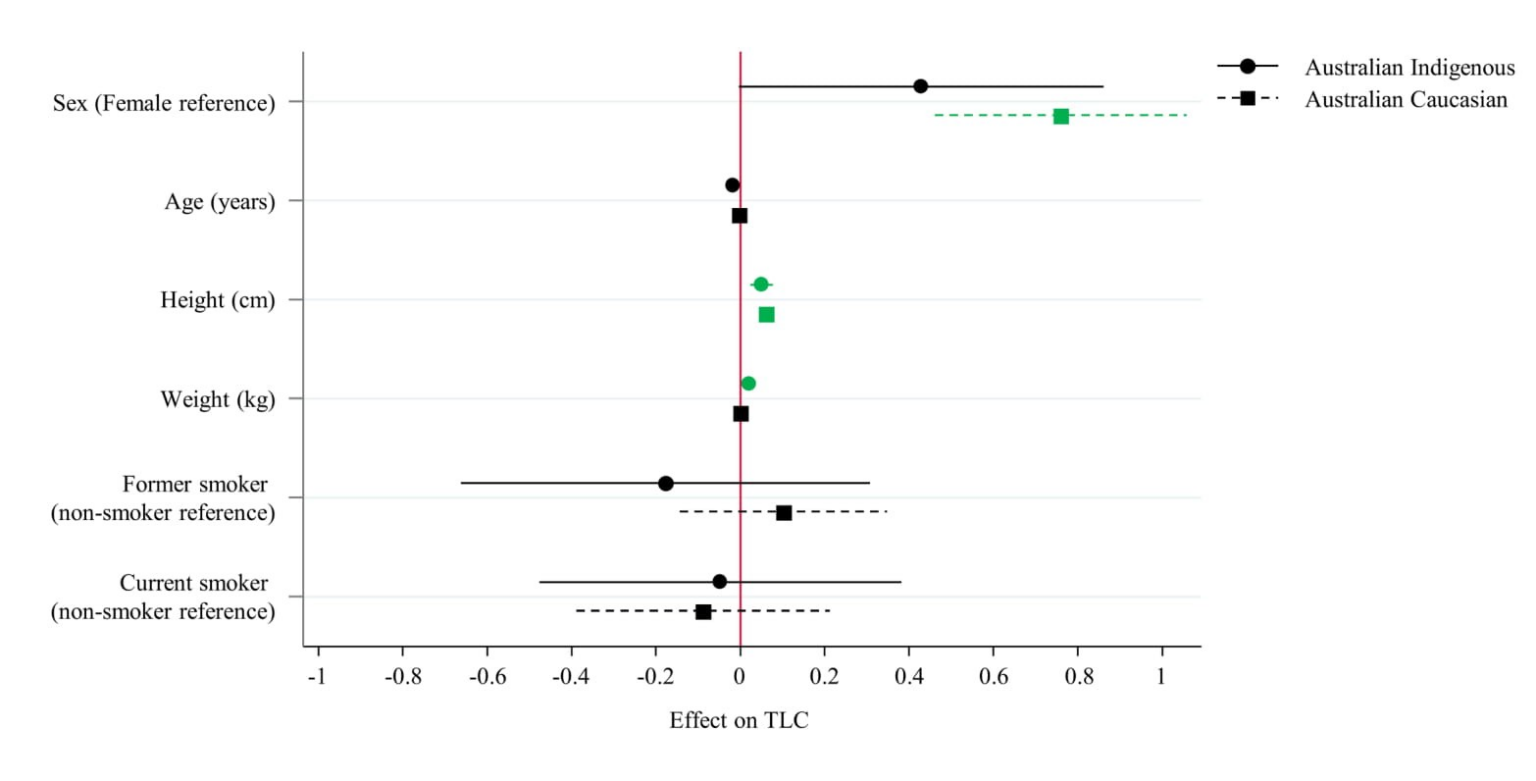
Coefficients plot of multivariate regression effects on Total Lung Capacity (TLC).

Significant differences were noted between the Indigenous Australian and Australian Caucasians in the multivariate regression models for multiple factors (Table 5). Sex bordered on significance for Indigenous Australians (p = 0.057), while it was strongly significant for Australian Caucasians (p<0.01). Weight however, displayed a significant association in the Indigenous Australian model (p = 0.05), and no association in the Australian Caucasian model (p = 0.94) (Fig 4). The overall fit of the models also showed significant difference, accounting for 34.7% of variance in outcomes among Indigenous Australians, and 58% of variance in Australian Caucasians.

**Table 5:** 

	Indigenous Australian		Australian Caucasian	
	Beta (95% CIs)	p-value	Beta (95% CIs)	p-value
**Sex (Female reference)**	0.41 (-0.01, 0.83)	0.057	0.76 (0.46, 1.06)	<0.01
**Age (years)**	-0.01 (-0.02, 0)	0.108	0 (-0.01, 0.01)	0.60
**Height (cm)**	0.05 (0.02, 0.07)	0.001	0.06 (0.05, 0.08)	<0.01
**Weight (kg)**	0.01 (0, 0.01)	0.050	0 (-0.01, 0.01)	0.94
**Former smoker (non-smoker reference)**	-0.2 (-0.67, 0.27)	0.401	0.1 (-0.14, 0.35)	0.41
**Current smoker (non-smoker reference)**	-0.07 (-0.49, 0.34)	0.726	-0.09 (-0.39, 0.21)	0.56
**Model R**^**2**^ **& p-value**	0.347	<0.001	0.580	<0.01

Data were expressed as means (95% confidence interval).

R^2^: adjusted form of Pearson’s product moment correlation coefficient.

Values fit within the ’Mild’ (0.3–0.5) or ’Moderate’ (0.5–0.7) correlation range.

## Discussion

This study demonstrated that Australian Indigenous people have significantly lower DLCO and TLC values for both absolute and percentage predicted values in both sexes and across ages in comparison to their Australian Caucasian counterparts. However, LLN values for DLCO and TLC did not demonstrate significant differences between the groups.

Recently, it has been recognised that ethnically diverse normative data for lung diffusing capacity and lung volumes needs to be a research priority for various ethnic populations around the world [38]. To the authors’ best knowledge, this is the first study to document LFPs for DLCO and TLC among Indigenous Australian people in comparison to the Australian Caucasian population, especially from the TEHS, NT region of Australia, where 30% of the population is of Indigenous Australian descent compared to the total 3.3% across Australia as a whole.

There have been only a few studies in the past illustrating the spirometry patterns among the Indigenous Australian population [39–43]. Some studies have demonstrated FEV_1_ and FVC values to be 20 to 30% lower [43], yet with a nearly preserved FEV_1_/FVC ratio [11, 14] among the Indigenous Australian population in comparison to non-Indigenous Australians. One previous study in the Australian setting has compared normative references for DLCO among middle aged Australians with other reference equations [44]. However, none of the aforementioned studies have explored the DLCO and TLC parameters among Indigenous Australian people in comparison to the Australian Caucasian cohort, including the recently published GLI normative data among Aboriginal or Torres Strait Islander children and young adults [45]. Hence, we believe that our study is of significant value to better understand the LFPs in the Indigenous Australian population, particularly in relation to DLCO and TLC parameters. Moreover, the GLI studies [46, 47] including “the GLI-2012 contemporary adult Australian spirometry study” [10], do not contain adequate representation of the Indigenous Australian people. Currently there are no well-established spirometry normative reference equations for the adult Indigenous Australian population and a recent study including children and adults with a smaller samples size has shown the GLI-2012 reference equation do not fit FEV_1_ or FVC for any other ethnic groups [48]. Similarly, our recently published study involving older adults demonstrated that the GLI-2012 norms do not fit for Australian Indigenous adults regardless of which ethnicity option was selected, including “others/mixed” [14].

The mechanisms underpinning the lower DLCO and TLC values observed in Indigenous cohort in this study in comparison to the Caucasian Australians are not entirely clear [13]. However, it may be reasonable to presume several genetic/intrinsic or extrinsic/environmental factors may be influential for such observation. These factors may include maternal smoking rates [26], lower birthweight [5, 27], increased childhood infections [6] and adolescent smoking [7, 8, 28]–aspects which have previously been observed to impact upon the applicability of normative values for a population [13, 49]. It is worth noting that Caucasian adults have typically shown significantly larger values for FVC compared to multiple different ethnic populations indicating the potential effect of evolutionary factors–thus the development of the GLI-2012 standard norms for multiple ethnicities [46]. Larger FVC values imply larger TLC values, though the latter is not always measured.

The prevalence of respiratory disorders is increasing worldwide, and in Australia, this is indeed true for the Indigenous population, imposing a significant burden on health care utilisation and cost [1–6, 11–14]. LFPs are crucial and integral in the diagnosis, management and monitoring of several respiratory disorders. Hence, it may be meaningful to make efforts to document normative LFPs not only in our Indigenous Australian people as in this study, but also in other underprivileged and vulnerable First Nations Indigenous populations around the world. The present study results may be of value to compare the findings to other ethnic groups and also to explore the fit to other ethnic Indigenous population. Moreover, in the absence of any comparative normative reference equation for DLCO and TLC in this reference population, the results of this study may indicate that LLN values may be a better indicator for clinical decision making in the Indigenous Australian population. However, further studies are warranted.

### Limitations

The results of this study must be viewed with caution. Our findings are applicable to the Indigenous Australian people living in the Top End NT of Australia and may not represent other Indigenous Australians living in other states and territory or other First Nations Indigenous people around the world. Moreover, the DLCO and TLC results represented in this study were performed on portable DLCO ndd device [33, 34, 50]. It is not clear if the results, particularly for TLC, would differ if the tests were performed using a body plethysmography device. However, measurement of DLCO and TLC values via body plethysmography would face significant challenges in this population, where 81% of Indigenous people reside in remote or very remote communities, many of which may be accessed only seasonally by light aircraft (S1 and S2 Figs) within a vast geographic area of 245,000 km^2^ (94,595.0 sq mi) and a population density of 0.16 people per kilometre, making such a study virtually infeasible [29, 30]. Therefore, the use of a portable DLCO ndd device is the most reasonable method to assess LFP’s for Indigenous subjects during respiratory specialist outreach visits to remote Indigenous communities (S3 Fig)—a unique model adopted for respiratory care in the TEHS region of NT of Australia [2, 3, 11]. The authors are also very aware and acknowledge that the sample size may be less than ideal for this study. However, given the fact that Australian Indigenous people have higher incidence of smoking [7, 8, 28, 45], are known to have a higher proportion of respiratory disorders [1–6] and experience geographic isolation, unprecedented challenges are imposed in recruiting study participants without pre-existing lung disease or smoking history for a study such as this.

## Conclusion

Despite some limitations and challenges, this is the first study to document the DLCO and TLC among Indigenous Australian people and compare it to Australian Caucasians. Indigenous Australians displayed significantly lower DLCO and TLC values for both absolute and percentage predicted in both sexes and across age ranges in comparison to their Australian Caucasian counterparts. However, the absolute LLN values for DLCO and TLC did not demonstrate significant differences between some groups and the LLN values may be a better predictor for clinical decision making. Further studies are however, warranted.

## Supporting information

S1 Fig Showing aerial view of a NT Australian Indigenous community. (PNG)

S2 Fig Showing a NT Australian Indigenous community airstrip. (PNG)

S3 Fig Showing the respiratory outreach team, including respiratory specialist physician, respiratory clinical nurse consultant, respiratory trainee medical officer, respiratory and sleep technologist and the pilot helping to unload the portable DLCO equipment. (PNG)

## Abbreviations

CI: Confidence Interval
DLCO: Diffusion capacity of carbon monoxide
FEV_1_: Forced Expiratory Volume in one second
FVC: Forced Vital Capacity
GLI: Global Lung function Initiative
HREC: Human Research Ethics Committee
LFPs: Lung function parameters
LLN: Lower limit of normal
NT: Northern Territory
PFT: Pulmonary function test
TEHS: Top End Health Service
TLC: Total lung capacity
