# Supplementary figures and images for: Comparison of diffusing capacity of carbon monoxide (DLCO) and total lung capacity (TLC) between Indigenous Australians and Australian Caucasian adults
Source: PLoS One. 2021 Apr 2;16(4):e0248900. doi: 10.1371/journal.pone.0248900 (PMC8018646; doi:10.1371/journal.pone.0248900)

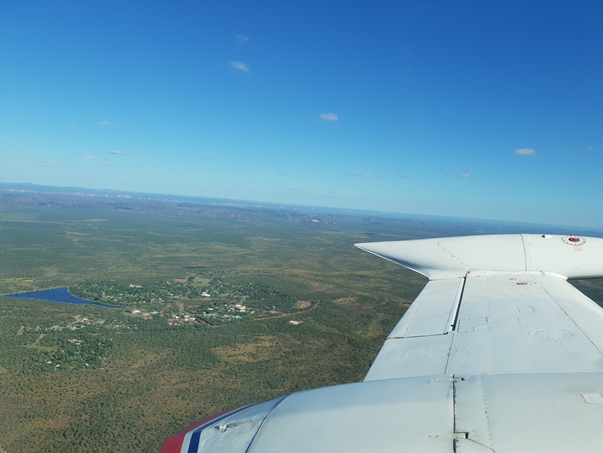

Supplement: S1 Fig — (PNG) [file pone.0248900.s001.png]

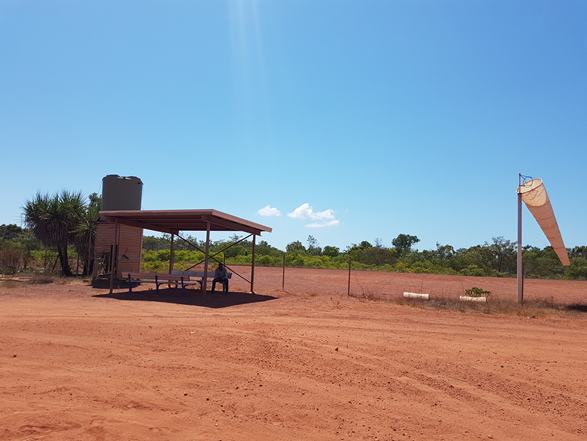

Supplement: S2 Fig — (PNG) [file pone.0248900.s002.png]

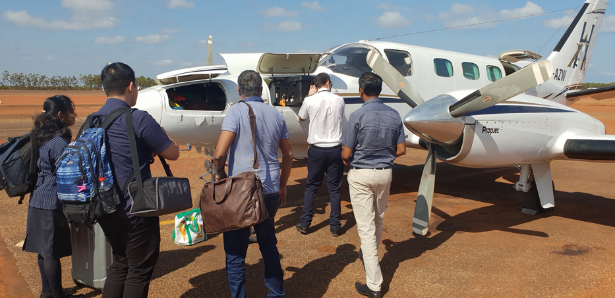

Supplement: S3 Fig — (PNG) [file pone.0248900.s003.png]
